# Who Rules over Migrants? Autocratic Tools in Migration Policies

**DOI:** 10.1007/s40803-026-00281-y

**Published:** 2026-08-20

**Authors:** Katharina Natter, Lieneke Slingenberg

**Affiliations:** 1 Institute of Political Science, Leiden University, Leiden, The Netherlands; 2 Faculty of Law, Vrije Universiteit Amsterdam, Amsterdam, The Netherlands

**Keywords:** Migration policy, Autocratization, Rule of law, Illiberalism, Policy tools

## Abstract

Across the world, political leaders – both democratic and autocratic –, are becoming more creative in mobilizing policy tools that circumvent judicial review or parliamentary oversight to enact migration policies in line with their economic, geopolitical or ideological goals. Such *autocratic policy tools*, as we call them, concentrate the power to make laws and rules in one hand, the hand that also executes the law. This is exemplified by straightforward ‘rule by decree’ situations, but also by the use of emergency powers or by the enactment of de facto policies that entirely lack a legal basis. The ten articles in this Special Issue bridge insights from Law and Political Science to examine such autocratic policy tools in migration policy across the globe. In this introduction, we (1) conceptualize the notion of *autocratic policy tools* and discuss implications for debates on autocratization and rule of law decline, (2) map the variety of such tools to showcase that they are mobilized by both democratic and autocratic regimes as well as for both restrictive and permissive goals, and (3) explore why autocratic policy tools are problematic by nature, irrespective of the content of the policy. Hereby, this Special Issue provides valuable insights for scholars to recognize those practices in other policy areas and to advance insights into autocratization and rule of law decay more broadly.

## Introduction

In 2013, a royal decree by Moroccan King Mohamed VI launched the country’s first-ever regularization of irregular migrants, followed by a series of ministerial circulars that expanded migrants’ socio-economic rights. While these policy changes were welcomed at the time by migrants, civil society and international actors, they were never enshrined in law, and thus at constant risk of being reversed at any point, dependent on executive will (Natter 2021). In 2022, the Dutch government introduced a 6-months waiting period for family members who were entitled to family reunification with recognized refugees. This new measure was explained in a letter to Parliament, and subsequently laid down in an internal policy document of the Immigration service, but lacked any legal basis (Slingenberg 2023). And in 2023, the European Commission signed an informal Memorandum of Understanding with the Tunisian government to increase migration control cooperation, bypassing the standard legal procedure that requires consultation of the European Council and Parliament (Natter 2023).

These instances are illustrative of the dynamics that we – co-editors of this Special Issue – encountered in our past research on migration policy. Coming from different disciplines, working on different regions of the world and engaged in distinct, yet as we will argue tightly connected scholarly debates, they prompted us to think collectively and systematically through what we call ‘autocratic policy tools’, that is: tools that concentrate the power to make laws and rules in one hand, the hand that also executes the law, without effective parliamentary and/or judicial oversight.

In this spirit, this Special Issue aims to bridge conversations in Law and Political Science to explore the variegated uses and the scope of such autocratic policy tools in migration policy. It asks: (1) What are the main defining features and effects of autocratic policy tools? (2) Are autocratic policy tools a prerogative of governments classified as autocratic or do democracies also mobilize autocratic policy tools? (3) How is the use of autocratic policy tools related to the substantive outcomes of policy reforms? In other words: Are autocratic policy tools mainly used to enact restrictive policy changes, limiting migrants’ rights; or can they also be a vehicle for permissive reform, increasing migrants’ rights?

As we elaborate below, these questions are intimately tied to scholarly debates in two fields which burgeoned in recent years, yet have remained surprisingly disconnected: scholarship on autocratization and democratic backsliding in Political Science and research around autocratic legalism and rule of law decline in Law. Empirically, the Special Issue zooms into migration as a policy area that is central to these political transformation processes, as it is considered to be a central element of the sovereignty of the state and touches on questions such as who can be a citizen of the country or where to draw borders between in- and out-siders (Tilly 1978; Torpey 2000; Wimmer 2002). Furthermore, migration law is often used by leaders as a field to experiment with policy practices that circumvent judicial or democratic scrutiny. Such policies affect not only the rights of migrants and refugees, but also of those working on and with them (like journalists or social workers; Kourakis 2025; Leroy 2025) and ultimately might spill over to the wider citizenry.

By zooming into how autocratic policy tools are used and work out in practice in the realm of migration policy, this Special Issue aims to provide valuable insights for scholars to recognize those practices in other policy areas and to better understand autocratization and rule of law decay more broadly. Beyond connecting debates in Political Science and Law, our examination of autocratic policy tools advances these debates by deliberately focusing on the *form* of law and policies, distinguishing it from an analysis of their (permissive/restrictive) *content* and the (democratic/autocratic) political regime *context* within which they are enacted. This, in turn, enables a normative analysis of the formal aspect of policy that problematizes the use of autocratic policy tools regardless of their substance and context.

In the following, we first provide our conceptualization of autocratic policy tools and outline how it allows to advance and connect ongoing debates in Political Science and Law around autocratization, autocratic legalism, democratic backsliding and rule of law decline. We then map the variety of autocratic policy tools used in migration policy around the globe, drawing on the ten Special Issue contributions. Together, they showcase that, against baseline assumptions, autocratic policy tools are used by both autocratic and democratic political regimes and for both restrictive and permissive purposes. We end with a discussion of why autocratic policy tools are problematic even if they increase migrant rights and with a call for a more integrated inter-disciplinary analysis of how the form of policymaking matters regardless of the policy substance at stake.

## Autocratic Policy Tools: Conceptualization and Implications

2025 marked the 8th consecutive year of global decline in rule of law (WJP 2025). At the same time, 45 states across all continents and home to almost 40% of the world population were undergoing autocratization (V-Dem 2023, 2025) – i.e. regime change away from democracy and towards autocracy (Cassani and Tomini 2020, p. 277; Lührmann and Lindberg 2019, p. 1096) – up from only 5 countries and 5% of the world population in 2011.

Within Law and Political Science, scholarship has proliferated over the past decade to circumscribe, understand and explain these developments. First of all, comparative politics debates have revolved around how to best define autocratization (Boese et al. 2021; Cassani and Tomini 2020; Lührmann and Lindberg 2019; Skaaning 2020; Tomini 2021). While Lührmann and Lindberg (2019, p. 1096) define autocratization in line with dominant negative understandings of autocracy as “any move away from democracy” that is a “substantial de-facto decline of core institutional requirements for electoral democracy”, we align with Cassani and Tomini (2019, p. 22) who define autocratization positively as “a process of regime change towards autocracy that makes the exercise of political power more arbitrary and repressive and that restricts the space for public contestation and political participation in the process of government selection. While rulers gain discretion and self-referentiality in the practice and maintenance of political power, citizens lose protection from it and control over it”.

Second, and relatedly, given the tendency in the literature to assume that autocracy and illiberalism go hand in hand, there has been a call to better distinguish both concepts analytically. Indeed, illiberalism has received renewed conceptual attention in recent years (Kauth and King 2020; Laruelle 2022; Sajó et al. 2021; Vormann and Weinman 2020; Waller 2024). This scholarship has shown that illiberalism can exist and thrive outside of autocratic regimes and that autocratic regimes are not by definition illiberal (Waller 2024). Also research around the notions of ‘liberal autocracy’ (Brumberg 2002; Cavatorta and Dalmasso 2009) and ‘illiberal democracy’ (Plattner 2020; Sajó 2021; Smilova 2021; Zakaria 1997) suggests that there are good reasons to differentiate both phenomena to better study how they interact, contradict or reinforce each other. Following Laruelle (2022), in this Special Issue we distinguish between autocracy as a configuration of institutions regulating how to assign and exercise political power and defined through the absence of core democratic institutions and processes, and illiberalism as a set of substantive claims, ideas and beliefs defined through the rejection of core tenets of liberalism. As we will show, distinguishing between autocratic institutions and policy tools on the one hand and illiberal (or restrictive) policy substance on the other hand is analytically fruitful for better understanding how autocratization works out in practice.^1^

Third, legal research has investigated how contemporary processes of autocratization are not typically accompanied by violent seizures of power, but unfold slowly, through technical changes from within the system. Scheppele, following Corrales, calls these kinds of processes “autocratic legalism”: autocratic leaders appeal to electoral legitimacy yet use legal tools available to consolidate power in few hands (Corrales 2015; Scheppele 2018). Such autocratic shifts, though not always lawful (Kazai 2024), are implemented through legal means. They are based on new interpretations of existing law and/or enshrined in new legislation. This provides legitimacy, and makes it harder to change or reverse these policies in the future. Accordingly, this scholarship aims to identify (early signs) of autocratization^2^ by analysing (changes in) the law (e.g. Graver and Gyöngyi 2024; Mieńkowska-Norkiene et al. 2024; Tóth 2019).

This Special Issue intervenes in and connects these debates by introducing the notion of ‘autocratic policy tools’. We define autocratic policy tools as tools that concentrate the power to make laws and rules within the executive, without effective parliamentary and/or judicial oversight. This entails e.g. the use of executive instruments such as decrees and ministerial directives, the use of emergency powers, de facto policies that lack a legal basis, or certain discretionary powers and exception frameworks. In that sense, autocratic policy tools can be both legal tools (e.g. decrees, regulations, laws) or soft law tools (ministerial guidelines, memoranda of understanding, policy rules, handbooks). These processes can be very visible for the general public, for instance when they are based on the formal declaration of a state of emergency or are accompanied by mass human rights violations. However, this is not always the case: Often, leaders aim to capture and exercise unconstrained power by using more subtle tools (Scheppele 2018).

To clarify: Policy tools such as decrees, exception frameworks or emergency powers are not autocratic by nature but can be (mis)used to infringe on the separation of power and to concentrate power within the executive by circumventing or removing either democratic control (i.e. parliamentary accountability) or judicial review or both. Such concentration of power and avoidance of accountability mechanisms can be explicit, for instance through the formal marginalization of the judiciary or parliament in decision-making processes, or implicit, such as in the case of obfuscation in policymaking processes, mis-information, opaque information environments or circumvention of consultation or other procedural requirements. In the latter case, autocratic policy tools also suffer from the absence of public scrutiny, in addition to the absence of parliamentary and judicial scrutiny.

This definition has three main conceptual implications: First, it disentangles the form of the policy from its content: autocratic policy tools can be used to enact both restrictive and permissive measures (i.e. lead to a decrease or increase in migrant rights). Second, it distinguishes the form of the policy from the broader political regime context in which it takes place: autocratic policy tools can be mobilized by governments and states classified along the entire democracy-autocracy spectrum. While regime dynamics might interact with the use of autocratic policy tools, this is not a deterministic relationship. Third, our definition focusses on tools that create, change and/or revoke policies and legislation and thus therefore does not include other kinds of power-concentrating tools, such as gerrymandering, the appointment of or changing the terms of office of judges, or changing the composition or mandate of independent institutions.

Ultimately, by distinguishing between context (political regime), form (policy tools) and content (substance of measures), this Special Issue makes two key contributions to ongoing debates in Political Science and Law: First, as we show empirically below, this distinction allows to identify and study the use of autocratic policy tools within both democratic and autocratic contexts, as well as for both restrictive and permissive goals. Second, it also allows to problematize autocratic policy tools in themselves and enables a normative analysis of the formal aspect of policy, regardless of its substance.

## Mapping Autocratic Policy Tools in Migration Policy

**Fig. 1:**
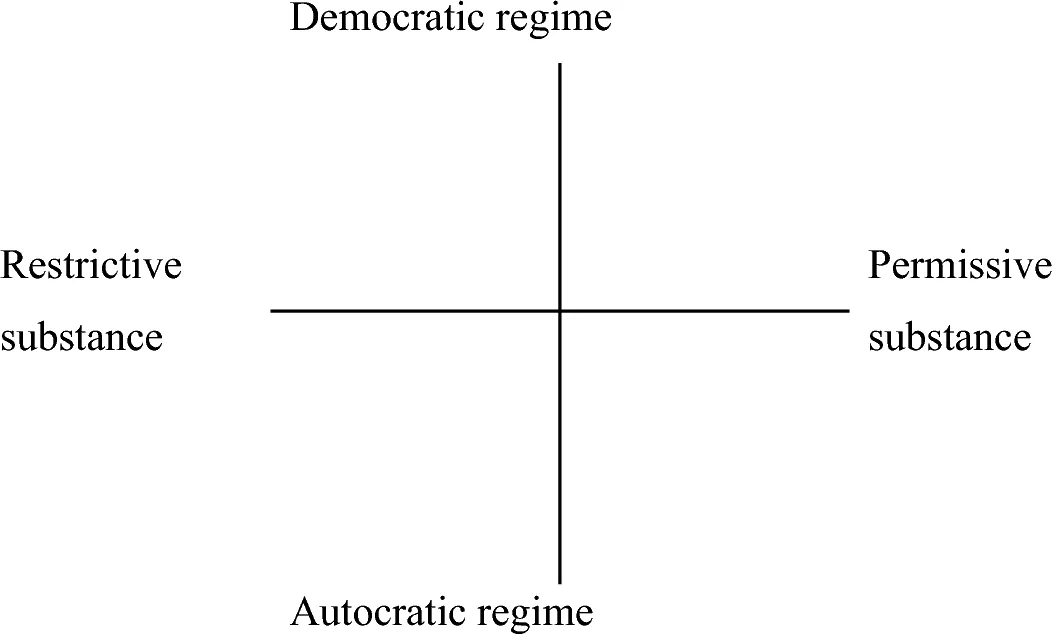
Spectrum of applicability of autocratic policy tools

The contributions in this Special Issue showcase the variety of autocratic policy tools and how they are used by democratic and autocratic regimes to enact both restrictive and permissive policy changes. The contributions provide examples of autocratic policy tools for each quadrant of this figure (Fig. 1):

The table below summarizes the variety of countries, autocratic policy tools, political regimes and policy substances covered by and used in the contributions of this Special Issue (Table 1):

**Table 1:** Overview of articles in the Special Issue

Author(s)	Countries/jurisdictions covered	Autocratic policy tool(s)	Political regime characteristics	Substance of change(s)
Bermejo-Casado and Iglesias	Spain	Rule by decree	Democratic context	Restrictive and permissive
Bornemann	EU	Discretion and sidelining of courts	Democratic context	Restrictive
Garcia Maia	Brazil	Rule by decree	Democratic and autocratic contexts	Restrictive and permissive
Hoppermann	EU, Türkiye & Lebanon	Bypassing parliamentary oversight and ad-hoc policies	Democratic and autocratizing contexts	Restrictive
Kaneti and Zhang	United States	Emergency powers and executive overreach	Democratic context	Restrictive
Malit	United Arab Emirates & Qatar	Outsourcing implementation and accountability	Autocratic contexts	Permissive
Nagel	Poland & Türkiye	Outsourcing implementation and accountability	Autocratizing context	Permissive
Riaz	United Kingdom	Sidelining of judicial oversight	Democratic context	Restrictive
Schotel	Belgium & The Netherlands	Manifestly illegal measures	Democratic context	Restrictive
Trompetero and Forero	Colombia	State of exception and rule-by-decree	Democratic context	Permissive

Thematically, the ten articles of this Special Issue can be organized in three groups. First, several contributions in this Special Issue examine the scope, function and effects of rule-by-decree as a central autocratic policy tool that weakens judiciary review and/or parliamentary scrutiny. They demonstrate that executive lawmaking is used by both autocratic and democratic leaders to achieve both permissive and restrictive policy aims. **Tainá Garcia Maia** retraces how the Brazilian executive has used power-concentrating tools to implement migration policy changes from the proclamation of the Republic in 1889 to 2023, a period that saw significant political transformations and regime changes. The analysis finds that rule by decree has been predominant across both democratic and autocratic governments, but that this has not per se resulted in the predominance of restrictive migration policy changes. **Ayesha Riaz** offers an in-depth case study of the Safety of Rwanda (Asylum and Immigration) Act 2024, showing how the Conservative Government under Rishi Sunak deliberately restricted judicial oversight and enabled executive-driven legislative dominance, hereby eroding legal accountability and the rule of law to introduce the Act. **Rut Bermejo-Casado and Juan Iglesias** analyse the interplay between executive power and democratic accountability in migration policymaking. They examine the frequent use of royal decree-laws in Spain since the 1980s and the conditions under which such policies support democratic goals and human rights as well as policies or undermine them. **María Gabriela Trompetero and Nelson Camilo Forero** examine how executive-driven policymaking can expand migrant rights within democratic regimes. Focusing on Colombia’s response to Venezuelan displacement (2017–2021), they show how autocratic policy tools—presidential decrees and administrative acts issued without legislative approval yet carrying immediate legal force – were used to extend protection to almost two million Venezuelans.

Second, the contributions by Bornemann, Kaneti and Zhang, as well asSchotel offer conceptual discussions and analysis of three types of autocratic policy tools – discretion, emergency powers and manifestly illegal policies – to identify their purpose and scope. **Jonas Bornemann** discusses discretion as a defining feature of migration policies. Through his analysis of EU migration law, specifically visa policies, resettlement, relocation and asylum decision-making, he introduces the analytical distinction between discretion as a historical relic of sovereign migration control within the EU and discretion as an autocratic tool that enables executive actors to strategically consolidate executive power and marginalise the role of courts. **Marina Kaneti and Xiaoqiao Zhang** argue that the use of emergency powers is not an aberration but a constitutive element of the liberal-democratic governance of mobility. By retracing mechanisms such as the Chinese Exclusion Laws, extraterritorial jurisdiction, and the plenary power doctrine in the case of the United States, they show that emergency powers and executive overreach have become normalized in modern liberal democracies. **Bas Schotel** then discusses manifestly illegal measures, i.e. measures that lack sufficient statutory basis or violate higher legal norms, as one form of autocratic policy tool. Engaging with research on bureaucratic law and legitimacy, he explores why democratic governments such as Belgium and The Netherlands might still choose to case manifestly illegal policies in the form of law, instead of operating completely outside the law.

Lastly, the contributions by Nagel, Hoppermann and Malit offer insights into how autocratic policy tools diffuse across actors by delegating the responsibility and accountability for implementation – be it to international organizations, sub-national actors or other states. **Max Nagel** disentangles the strategies of autocratizing governments when adopting permissive migration policies. Zooming into Poland and Türkiye, he shows that autocratizing governments satisfy employer demand for labor while avoiding backlash from migration-skeptical voters by employing “responsibility dumping”, i.e. by shifting the responsibility and accountability for permissive migration policy to subnational and non-state actors without granting them sufficient resources. **Froilan Malit** analyses how states in the Gulf (UAE and Qatar) introduce permissive migration reforms despite their autocratic regime orientations. He shows that these regimes employ a range of autocratic policy tools such as joint technical agreements, ministerial decrees, and resolutions in order to co-opt international organizations to implement pro-migrant rights reforms, hereby advancing their domestic and geopolitical objectives. Finally, **Judith Hoppermann** examines the diffusion of autocratic policy tools in the context of EU border externalization to the Middle East. She shows how the EU’s use of autocratic tools in their migration policy, specifically the bypassing of parliamentary oversight to conclude migration deals, incentivizes the adoption of autocratic migration tools such as ad-hoc, ambiguous policies by Türkiye and Lebanon.

Together, the collection of articles gives insight into the variety, scope and effects of autocratic tools in the field of migration policy. They also examine the role of different actors – not only the executive, but also judicial, diplomatic and societal actors – facilitating, maintaining or potentially resisting the use of autocratic policy tools. Ultimately, distinguishing between the form and content of policy tools also enables a normative analysis of the formal aspect of autocratic policy tools, which is obscured as long as autocratic policy tools are assumed to be automatically tied to restrictive substance. This is what we turn to now.

## Why Autocratic Policy Tools Are Problematic by Nature

This Special Issue shows that autocratic policy tools do not automatically lead to restrictive policies. However, this does not imply that they should be condoned or celebrated when they lead to rights expansions. In fact, autocratic policy tools are problematic in their own right, regardless of the content of the policy they are advancing, as they by nature imply the absence or limitation of external constraints on power. This infringes on a fundamental kind of freedom: freedom as non-domination.

The concept of freedom as non-domination has its origins in the classical republican tradition, but has recently received renewed interest by, what is often called, ‘neo-republicanism’ (Lovett 2010; Pettit 1997, 2012). Freedom in terms of non-domination is about the absence of mastery by others and can, therefore, be contrasted with both freedom as non-interference (negative freedom) and freedom as self-mastery (positive freedom) (Pettit 1997 p. 22). In neo-republican theory, ‘domination’, is understood as subjection to a threat of unconstrained interferences. Domination is the kind of power no one should be subjected to, even if this power is not being used or only used for legitimate reasons (Lovett and Pettit 2009; Lovett 2016). This more collective form of freedom focuses on the structural aspects of a power relationship, rather than on the actual outcomes of the exercise of power (Jacobs 2022). The standard example used in this literature is the situation of slavery. Even if a slave-owner is a benevolent master who does not obstruct the enslaved person’s individual choices, we would still explain the situation as a situation of fundamental unfreedom. This is because of the structural aspects of the relationship: the master can change his mind and behavior at any time, his power is not externally constrained.

Autocratic policy tools aim to concentrate the power to make and amend law and policies within the executive and limit external checks and balances. Accordingly, they increase the possibility of being subjected to dominating power, which harms personal autonomy, human dignity and self-respect (Lovett 2022, pp. 74–75; Jacobs 2022, p. 73). This is also true for policies that increase individual rights and protection, as they can be made highly conditional and selective and/or can be repurposed and used for other aims at any moment. For instance, as Garcia Maia shows in her historical analysis of Brazilian immigration policies in this Special Issue, even when leading to migrant right expansions, the use of autocratic policy tools entails the risk of maintaining or even reinforcing relations of domination, marginalization and ethnic selection in practice. Similarly, Trompetero and Forero show that also executive decrees used to expand refugee rights in Colombia generated fragile legality, which makes these protections temporary, reversible, and dependent on executive will. More generally, once the use of autocratic policy tools is established and normalized – even if for rights-expansive policies –, they can be quickly repurposed and used for other aims or target populations. Indeed, Scheppele warned that implementing autocratic shifts through the law can happen surprisingly easily, because the autocratic leader is ‘simply taking in the political harvest others have planted’ (Scheppele 2018, p. 569).

Concentrating and consolidating power within the executive, through the use of autocratic policy tools, is particularly problematic in the field of migration law. Migrants are highly dependent on the executive, for fundamental aspects of their lives. Migration law covers deprivation and restriction of liberty (detention and containment); access to employment, social assistance and housing; and family and private life. In short, migration law decides where and with whom migrants can live and work (Family 2012). For nationals, the decision as to where and with whom they can live and work is usually not determined by law. And if so, this is mainly done by civil (family or labour) or criminal law. In those fields of law, it is usually the judge who decides in individual cases, not the executive. Accordingly, as compared to nationals, migrants’ are subjected to executive power to a much larger extent. A high level of dependency makes subjection to arbitrary power more problematic, requiring stronger constraints (Lovett 2010/2016; Slingenberg 2020).

Disentangling the form of policies from their content, as we do in the Special Issue, therefore provides not only more analytical clarity to study the variety and complexity of migration policy, but also a way to normatively think through the systemic dangers that the normalization of autocratic policy tools entails for democracy and rule of law more broadly, and for migrants in particular.

## Data Availability

No datasets were generated or analysed during the current study.

## Footnotes

1 In this introduction, we will use the term ‘restrictive’ to refer to policies that limit migrants’ rights and the term ‘permissive’ to refer to policies that increase migrants’ rights. Restrictive policies might be illiberal policies if they violate core aspects of liberalism, such as fundamental rights, but can also stay within the boundaries of liberalism. For that reason, we prefer to use the terms ‘restrictive’ and ‘permissive’ from now on, which are more descriptive in nature.

2 Other terms are also used, such as ‘rule of law decay’ (e.g. Adams and Janse 2019), ‘rule of law backsliding’ (e.g. Pech and Scheppele 2017) or (mostly in the US context) ‘executive overreach’ (e.g. Scheppele and Pozen 2020). They all include processes of power concentration within the executive, through the shifting of power and limiting checks and balances.
